# SLFN11 Negatively Regulates Noncanonical NFκB Signaling to Promote Glioblastoma Progression

**DOI:** 10.1158/2767-9764.CRC-22-0192

**Published:** 2022-09-13

**Authors:** Mariafausta Fischietti, Frank Eckerdt, Ricardo E. Perez, Jamie N. Guillen Magaña, Candice Mazewski, Sang Ho, Christopher Gonzalez, Lukas D. Streich, Elspeth M. Beauchamp, Amy B. Heimberger, Aneta H. Baran, Feng Yue, C. David James, Leonidas C. Platanias

**Affiliations:** 1 Robert H. Lurie Comprehensive Cancer Center of Northwestern University, Chicago, Illinois.; 2 Division of Hematology/Oncology, Department of Medicine, Feinberg School of Medicine, Northwestern University, Chicago, Illinois.; 3 Department of Neurological Surgery, Feinberg School of Medicine, Northwestern University, Chicago, Illinois.; 4 Department of Pathology, Feinberg School of Medicine, Northwestern University, Chicago, Illinois.; 5 Department of Medicine, Jesse Brown Veterans Affairs Medical Center, Chicago, Illinois.; 6 Department of Biochemistry and Molecular Genetics, Feinberg School of Medicine, Northwestern University, Chicago, Illinois.

## Abstract

Glioblastoma (GBM) is an aggressive and incurable brain tumor in nearly all instances, whose disease progression is driven in part by the glioma stem cell (GSC) subpopulation. Here, we explored the effects of Schlafen family member 11 (SLFN11) in the molecular, cellular, and tumor biology of GBM. CRISPR/Cas9-mediated knockout of *SLFN11* inhibited GBM cell proliferation and neurosphere growth and was associated with reduced expression of progenitor/stem cell marker genes, such as *NES*, *SOX2*, and *CD44*. Loss of *SLFN11* stimulated expression of NFκB target genes, consistent with a negative regulatory role for SLFN11 on the NFκB pathway. Furthermore, our studies identify p21 as a direct transcriptional target of NFκB2 in GBM whose expression was stimulated by loss of *SLFN11*. Genetic disruption of *SLFN11* blocked GBM growth and significantly extended survival in an orthotopic patient-derived xenograft model. Together, our results identify SLFN11 as a novel component of signaling pathways that contribute to GBM and GSC with implications for future diagnostic and therapeutic strategies.

**Significance:**

We identify a negative regulatory role for SLFN11 in noncanonical NFκB signaling that results in suppression of the cell-cycle inhibitor p21. We provide evidence that SLFN11 contributes to regulation of stem cell markers in GBM, promoting the malignant phenotype. In addition, SLFN11 targeting triggers p21 expression and antitumor responses. Our studies define a highly novel function for SLFN11 and identify it as a potential therapeutic target for GBM.

## Introduction

Glioblastoma (GBM), classified as World Health Organization grade 4 glioma (1), is the most frequent primary tumor in the brain, with a 5-year survival estimate of only 7% (2). Maximal safe surgical resection, followed by chemoradiation and adjuvant temozolomide (TMZ) treatment has been the standard of care in treating GBM for the past 17 years (3). However, this treatment approach results in modest survival benefit, with 14.6 months being the median value (3). Tumor cell dissemination in normal brain tissue and extensive subpopulation heterogeneity are hallmarks of GBM (4, 5), and are key factors in the relative lack of success in treating this cancer. Isocitrate dehydrogenase (IDH) mutational status is an important prognostic factor for patients with GBM, with IDH wild-type (WT) patients exhibiting a lesser median survival compared with patients with IDH mutant tumor (6). As such, GBM is now genetically defined as IDH WT versus mutant (1). Transcriptomic analysis has also been used in the subclassification of GBM, with mesenchymal, proneural, and classical tumors being widely accepted as transcriptionally defined subtypes (7, 8). Thus far, there has been no success in identifying therapeutics that are effective in controlling genetically nor transcriptionally defined GBM subtypes. As such, there is a compelling need for increased understanding of GBM molecular biology which may reveal therapeutically actionable targets for treating GBM.

Previously, we described Schlafen 5 (SLFN5) as a potential biomarker and therapeutic target in GBM by demonstrating that elevated SLFN5 expression promotes GBM malignant phenotypes (9). This implicated, for the first time, the family of *Schlafen* (SLFN) genes in the pathogenesis of GBM. SLFN genes were initially identified for their ability to induce a reversible G_0_–G_1_ cell-cycle arrest in thymocytes (10). SLFNs are found in vertebrates from frogs to mammals with variable homology across species (11).

Previous studies have established SLFNs as IFN-responsive genes, with implications in cell differentiation, proliferation, immune cell regulation (10), and as inhibitors of viral replication (12). Recent studies have explored roles for human SLFN family members in cancer biology (reviewed in ref. 13) and found that the contributions of SLFNs in the regulation of oncogenic processes are complex. On one hand, SLFN5 overexpression suppresses breast tumor growth in mice and elevated SLFN5 expression correlates with better survival in breast cancer (14) and renal cell carcinoma (15). In addition, SLFN5 knockdown increases transformation and invasion in malignant melanoma (16). On the other hand, SLFN5 is highly expressed and contributes to tumor progression in pancreatic ductal adenocarcinoma (17), castration-resistant prostate cancer (18), and gastric carcinoma (19). This indicates that SLFNs can have diverse and, sometimes, opposing functions in cancer, possibly in a tissue-specific manner. A similarly complicated role can be assumed for SLFN11, which was found to exhibit a broad range of expression in a The Cancer Genome Atlas (TCGA) pan-cancer dataset (20), and the NCI-60 cancer cell line panel (21, 22). Also, SLFN11 is highly expressed in some cancers, such as Ewing sarcoma, pediatric sarcomas, mesothelioma, and renal cell carcinoma, while its expression is low in other types of cancer such as tumors of the ovary and pancreas (20, 22–24).

In this work, we provide evidence for SLFN11 contributing to GBM cell proliferation, neurosphere growth, and expression of progenitor/stem cell markers. We demonstrate that SLFN11 associates with components of the NFκB family of inducible transcription factors that are involved with the regulation of numerous cellular processes (25). Activation of the noncanonical NFκB pathway is stimulated by regulated processing of p100 into the DNA-binding transcription factor p52, which either homodimerizes (p52:p52) or heterodimerizes with RelB (p52:RelB) to modulate transcription of a plethora of target genes (26). Using immunoprecipitation (IP) mass spectrometry analysis, we found association of SLFN11 with NFκB2 in GBM. Genetic disruption of *SLFN11* stimulated expression of NFκB target genes, including *CDKN1A* (p21) and significantly delayed tumor growth and improved survival in a GBM orthotopic patient-derived xenograft (PDX) mouse model.

## Materials and Methods

### Cell Lines

All cell lines were incubated in a 37°C humidified incubator with 5% CO_2_ and were grown in DMEM supplemented with 10% FBS. U87 cells were kind gift from Dr. Alexander Stegh, LN229 cells from Dr. Chi-Yuan Cheng, and GBM6 PDX from Dr. C. David James (all Northwestern University, Chicago, IL). All cell lines were tested for *Mycoplasma* every 4 months and every 6 months cell lines were tested by short tandem repeat analysis and authenticated using published reference databases.

### Three-dimensional Culture of GBM Cell Lines and PDX Cells and Neurosphere Assay

GBM cell lines and GBM6 PDX cells were grown under cancer stem cell (CSC) culture conditions in three-dimensional (3D) and plated for neurosphere assays as described previously (27). GBM6 PDX line, stably expressing Luciferase was described previously (28). Neurosphere assay was performed as in ref. 29, and neurosphere cross-sectional area was determined as described before (30).

### Orthotopic Tumor Xenograft Model

All animal studies were carried out under an approved protocol of the Institutional Animal Care and Use Committee (IACUC) of Northwestern University (Chicago, IL). Luciferase-expressing GBM6 cells were suspended in RPMI at a concentration of 1.5 × 10^5^ cells per μL. Anesthetized female homozygous NCr nude mice (5–6 weeks; Taconic) were placed on a heating pad, the surgical area was cleaned with 70% ethanol and betadine solution. A para-sagittal skin incision was made (∼10 mm) over the middle frontal to parietal bone. The exposed skull surface was treated with 3% hydrogen peroxide solution and a 25-gauge needle was used to create a burr hole 2 mm lateral right of the bregma and 1 mm posterior to the coronal suture. GBM6 WT and *SLFN11* knockout (KO) cells (2 μL cell suspension) were implanted through a Hamilton syringe over a period of 1 minute. After 1 additional minute, the syringe was slowly withdrawn, and the wound was closed with staples. Mice received postsurgical care according to IACUC guidelines and were imaged weekly by bioluminescence imaging using a Lago/Lago X—Spectral Instruments Imaging system as described previously (17).

### Generation of Doxycycline-inducible SLFN11 Overexpression Cell Line

LN229-TetON-SLFN11-Myc-Flag stable cell lines were generated as described previously (17).

### Generation of *SLFN11* KO Cell Lines Using CRISPR/Cas9 Technology

U87 were transfected using Turbofect (Thermo Fisher Scientific) with Cas9, single-guide RNA (sgRNA) targeting the *SLFN11* gene, and homology direct repair plasmids. Cells were kept under Puromycin selection for 2 weeks and then sorted for high expression of red fluorescent protein using FACS. LN229 and GBM6 cells were transduced with pLVX-hEF1α-Cas9-Blast and pLVX-CMV-SLFN11 sgRNAs-PURO using TransDux MAX Lentivirus Transduction enhancer (System Biosciences) according to the manufacturer's instructions. Cells were kept under Puromycin selection. LN229-Cas9 cells were used as control. LN229 *SLFN11* KO cells were seeded as single cells in 96-well plates by FACS for generation of single clones. The clone showing the most efficient KO was used for experiments in this study.

### Generation of Flag-SLFN11 Constructs and SLFN11-overexpressing Cells

pLenti-CMV-Hygro-SLFN11-Flag plasmid was generated as described previously (17). U87 and LN229 cells reexpressing SLFN11 (*SLFN11* KO+SLFN11) were generated using lentiviral transduction as described above.

### Hematoxylin and Eosin Staining and IHC Staining

Mouse brains with GBM6 *SLFN11* WT and KO tumors were collected and processed for hematoxylin and eosin (H&E) staining and IHC staining by the Human Pathology Core of Northwestern University as described previously (30). Slides were analyzed and scored for p21 expression by a board-certified pathologist Dr. Lukas D. Streich. Slides were scanned using Hamamatsu NanoZoomer Digital slide scanner and images were exported using NDP.view2 Viewing software.

### Cell Proliferation Assays

*SLFN11* WT and KO U87 and LN229, and KO+SLFN11 U87 and LN229 cells, and *SLFN11* KO siCTRL/si*CDKN1A* U87 and LN229 cells were plated in 6-well plates in duplicate (50,000 cells/well). Cells were dissociated by Trypsin and counted after 2, 4, and 7 days using TC20 Automated Cell Counter (Bio-Rad).

### Cell Lyses and Immunoblotting

Cell lysis and immunoblotting were performed as described previously (31). Chemiluminescence was detected using a ChemiDoc MP imager or autoradiography film. Films were digitally scanned with Adobe Photoshop using a Canon CanoScan 8800F scanner.

### Real Time qPCR

*SLFN11* WT and KO U87, LN229, and *SLFN11* KO+SLFN11 U87 and LN229, and GBM6 cells were lysed in RLT buffer (Qiagen). Tumors from mice bearing *SLFN11* WT and KO GBM6 were homogenized in RLT buffer using TissueRuptor (Qiagen). Total RNA was isolated using RNeasy minikit (Qiagen) and retrotranscribed using High Capacity cDNA reverse transcription kit (Applied Biosystems). TaqMan qRT-PCR was performed using SsoAdvanced Universal Probes Supermix (Bio-Rad) according to the manufacturer's instructions, using Taqman probes (see Supplementary Table S1).

### Co-IP Assays

Cells were lysed with NP-40 lysis buffer (40 mmol/L HEPES pH 7, 120 mmol/L NaCl, 1 mmol/L EDTA, 10 mmol/L Na Pyrophosphate, 50 mmol/L NaF, 10 mmol/L β-glycerophosphate and 0.1% NP-40). FLAG-M2–conjugated sepharose beads (Sigma-Aldrich) and Myc-Tag–conjugated sepharose beads (Cell Signaling Technology) were used for IP as described previously (17).

### Proteomics IP Analysis Using LC/MS-MS

LN229-TetON-SLFN11-Myc-Flag cells were plated in 150 mm dishes. The following day, cells were either left untreated or treated with doxycycline (DOX) for 48 hours. Subsequently, cells were either left untreated or irradiated with 8 Gy for 30 minutes. Cell lysates were prepared in NP-40 lysis buffer and then IP was performed using Myc-Tag–conjugated sepharose beads (Cell Signaling TEchnology). No DOX-treated samples were used as negative controls. Samples were then processed and analyzed as described previously (17).

### Gene Annotation and Protein Function Enrichment Analysis

Protein lists identified in LC/MS-MS were converted to gene lists and were submitted to the Metascape database, a gene annotation and analysis resource (http://metascape.org/), for pathway and process enrichment analysis as described previously (17).

### Bioinformatics Analysis

TCGA_GBM gene expression data using the RNA sequencing (RNA-seq) platform or the Agilent (Agilent-4502A) array were analyzed using the GlioVis portal (http://gliovis.bioinfo.cnio.es/; ref. 32).

### Immunofluorescence

*SLFN11* WT and KO U87 and LN229 cells were plated on coverslips in 12-well plates (25,000 cells/well). After 5 days, cells were washed with PBS and fixed with 4% paraformaldehyde (PFA) for 30 minutes. For permeabilization, cells were incubated with PBS+0.1% Triton for 20 minutes at room temperature. Subsequently, cells were washed and incubated with blocking buffer (2% BSA+0.1% Triton in PBS) for 50 minutes at room temperature. Cells were then incubated with anti-p21 primary antibody (Cell Signaling Technology) overnight at 4°C. The next day, cells were washed and sequentially stained with AlexaFluor546-phalloidin and 4ʹ,6-Diamidine-2ʹ-phenylindole dihydrochloride (DAPI). After five washes, coverslips were mounted on microscope slides using ProLong Gold Antifade Mountant (Thermo Fisher Scientific). Images were acquired using a Leica DMi8 inverted microscope with objective lens 20× air Plan Fluotar, NA 0.40. Cells positive for p21 were counted manually using Fiji-ImageJ software.

### Confocal Laser Scanning Microscopy

Microscopy was performed using a Nikon A1plus inverted microscope. Objective lens was from Nikon: 20× air Plan Apo objective, NA 0.75. Fluorochromes were from Invitrogen and included AF488 (green) and AF546 (red). DAPI (blue) was from Roche. For microscopic analysis, the acquisition software NIS Elements (Nikon) was used.

### siRNA-mediated Knockdown

Control and *NFKB2-*targeting siRNAs were from Dharmacon, Control and *CDKN1A* were from Santa Cruz Biotechnology and used with Lipofectamine RNAiMAX reagent and Opti-MEM medium (Thermo Fisher Scientific), as described previously (30).

### Chromatin IP

Cells were grown as 3D Neurospheres. Chromatin immunoprecipitation (ChIP) was performed using the SimpleChIP Enzymatic Chromatin IP Kit with Magnetic Beads from Cell Signaling Technology, as per the manufacturer's instructions. Antibodies for NFκB2 p100/p52 and RelB were purchased from Cell Signaling Technology. Normal rabbit IgG was used as a negative control. qRT-PCR was performed on purified immunoprecipitated DNA for the *CDKN1A* promoter (the *RPL30* promoter served as a negative control) using SsoAdvanced Universal SYBR Green Supermix (Bio-Rad) according to the manufacturer's instructions. All qRT-PCR signals were normalized to the input DNA.

### Primer Design Strategy for ChIP

To determine potential NFκB binding sites on the *CDKN1A* promoter, 3,000 bp upstream and 100 bp downstream from the transcription start site were analyzed using the JASPAR database (33) at a relative profile score threshold of 80% for known human NFκB binding motifs.

### 3D Matrigel Invasion Assay

3D tumor cell invasion was determined using the Cultrex 3D Spheroid Cell Invasion Assay (Trevigen) as described previously (9).

### Statistical Analysis

All statistical analyses were performed using GraphPad Prism 8.0 and *P* values <0.05 were considered statistically significant.

### Data Availability

The data generated in this study are available upon request from the corresponding author. The mass spectrometry proteomics data have been deposited to the ProteomeXchange Consortium via the PRIDE partner repository with the dataset identifier PXD033913.

## Results

### *SLFN11* Expression is Elevated in GBM and Associated with Poor Survival

**FIGURE 1:**
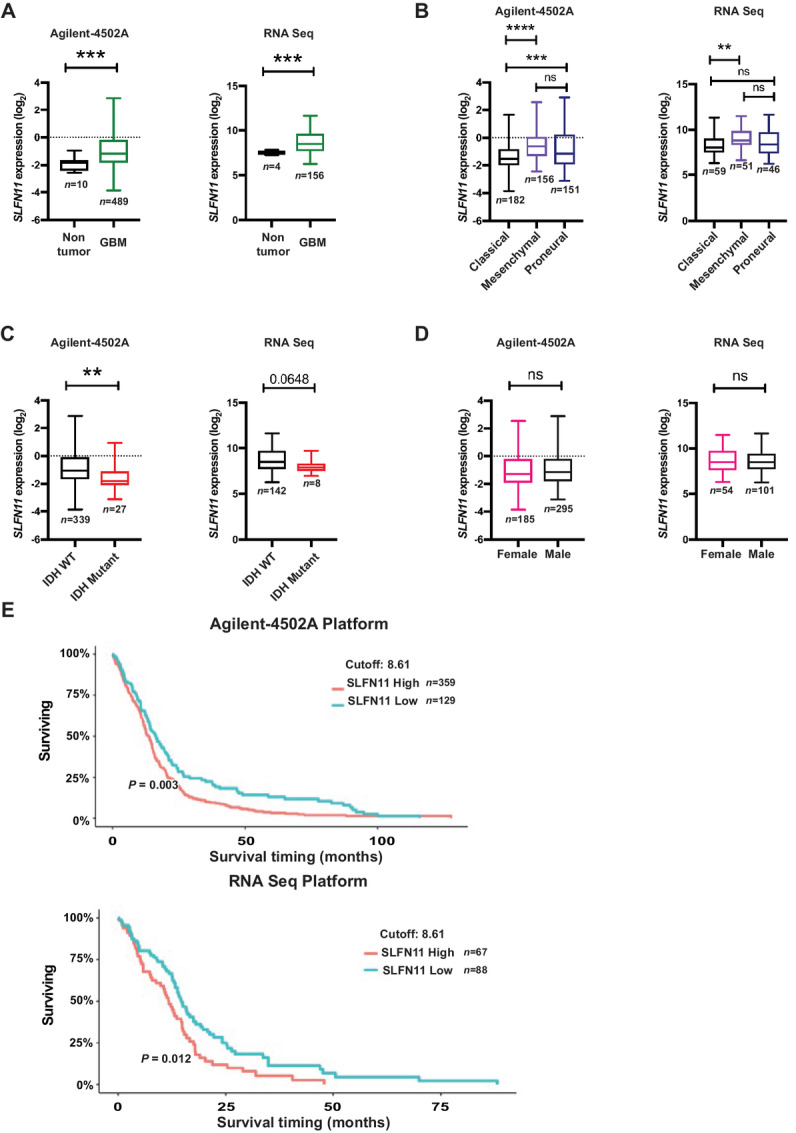
*SLFN11* is overexpressed in GBM and high *SLFN11* expression correlates with poor overall survival in patients with GBM. **A–D,***SLFN11* gene expression data from TCGA_GBM dataset were downloaded from the GlioVis portal (http://gliovis.bioinfo.cnio.es/) from the Agilent (Agilent-4502A) array (left) or the RNA-seq platform (right) and analyzed using GraphPad Prism 8. Expression of *SLFN11* plotted in nontumor and GBM (**A**), GBM subtypes (**B**), IDH status (**C**), and gender (**D**). (**A**, **C**, **D**) Unpaired *t* test with Welchs’ correction. **B,** Ordinary one-way ANOVA with Tukey multiple comparison test; **, *P* < 0.01; ***, *P* < 0.001; _****_, *P* < 0.0001. **E,** TCGA_GBM survival data from the Agilent-4502A array and the RNA-seq platform were analyzed by Kaplan–Meier curve comparison with GlioVis portal (http://gliovis.bioinfo.cnio.es/) using optimal cutoff. Optimal cut-off points were designated by GlioVis database.

Using the Sun and Rembrandt datasets that were available through the Oncomine database, we previously found elevated SLFN11 expression in GBM and this was associated with worse prognosis (9). To corroborate and extend these findings, we now interrogated TCGA dataset (TCGA_GBM) using the GlioVis portal (http://gliovis.bioinfo.cnio.es/; ref. 32). Results from microarray (Agilent-4502A) as well as RNA-seq revealed that *SLFN11* expression is significantly elevated in GBM, as compared with normal brain tissue (Fig. 1A). Among transcriptionally defined subtypes of GBM, *SLFN11* expression is higher in the mesenchymal subtype relative to classical and proneural subtypes (Fig. 1B). With regard to GBM genetic subclassification, *SLFN11* is higher in IDH WT than IDH mutant tumors (Fig. 1C). There is no significant gender-associated difference in *SLFN11* expression (Fig. 1D), but there is clear indication of increasing *SLFN11* expression being associated with decreasing GBM patient survival (Fig. 1E).

### Genetic Disruption of *SLFN11* Impairs GBM Cell Growth, Which is Rescued by Exogenous *SLFN11* Expression

**FIGURE 2:**
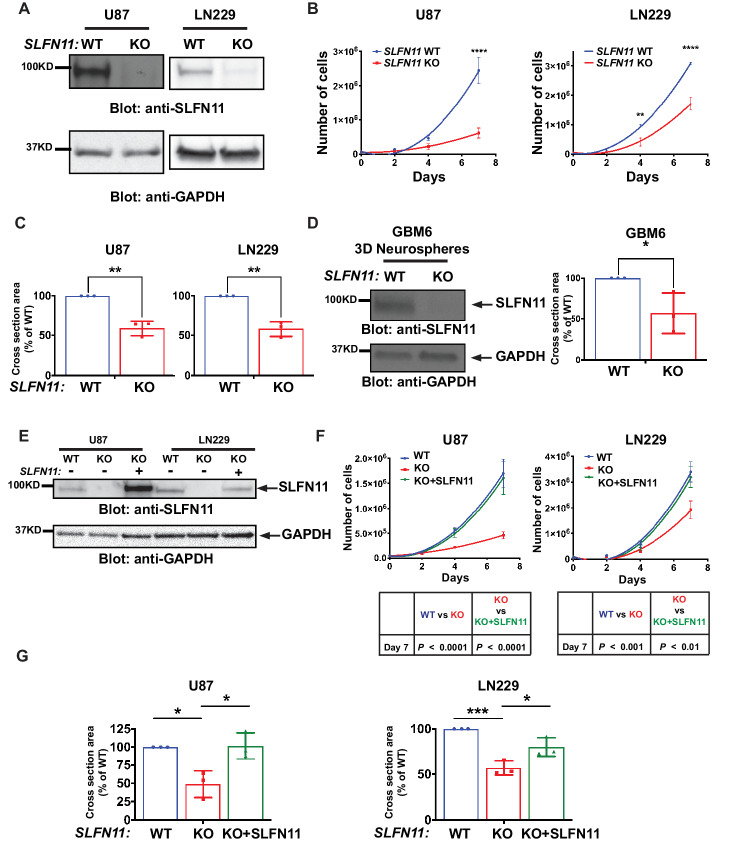
Loss of *SLFN11* inhibits proliferation and neurosphere growth in GBM cell lines and PDX neurospheres. *SLFN11* KO U87 and LN229 cells were generated using CRISPR/Cas9 technology. **A,** Protein expression of SLFN11 in WT and *SLFN11* KO U87 (left) and LN229 (right) cells was determined by Western blot analysis. Equal amounts of total cell lysates from the indicated cells were resolved by SDS-PAGE and immunoblotted with the indicated antibodies. **B,***SLFN11* WT and *SLFN11* KO U87 and LN229 cells were plated in 6-well plates and counted at days 2, 4, and 7 after seeding. Data are means of number of cells ± SEM of three independent experiments, each done in duplicate. Two-way ANOVA with Sidak multiple comparison test; **, *P <* 0.01; ****, *P <* 0.0001. **C,***SLFN11* WT and *SLFN11* KO U87 (left) and LN229 (right) cells were plated into round bottom 96-well plates under CSC culture conditions to form 3D neurospheres. After 7 days, spheres were imaged using a Cytation 3 Cell Imaging Multi-Mode Reader to determine cross-sectional area. Data are expressed as percentages of WT parental cells and represent means ± SEM of three independent experiments, each done in triplicate. Two-tailed unpaired *t* test; **, *P* < 0.01. **D,***SLFN11* KO GBM6 cells were generated using CRISPR/Cas9 technology. Expression of SLFN11 was determined by Western blot analysis (left) as described above. *SLFN11* WT and KO GBM6 cells were plated into round bottom 96-well plates under CSC culture conditions to form 3D neurospheres. After 7 days, spheres were imaged using a Cytation 3 Cell Imaging Multi-Mode Reader to determine cross-sectional area (right). Data are expressed as percentages of WT parental cells and represent means ± SEM of three independent experiments, each done in triplicate. Two-tailed unpaired *t* test; *, *P* < 0.05. **E,** U87 and LN229 *SLFN11* KO cells were stably transduced with FLAG-SLFN11-pLenti. Expression of SLFN11 in U87 (left) and LN229 (right) cells was monitored by immunoblotting as described above. **F,***SLFN11* WT, *SLFN11* KO and *SLFN11* KO+SLFN11 U87 (left) and LN229 (right) cells were plated in 6-well plates. After 2, 4, and 7 days, cells were counted. Data are means of number of cells ± SEM of three independent experiments, each done in duplicate. Statistical analysis was performed using a two-way ANOVA with Sidak multiple comparison test for U87 (bottom left) and LN229 (bottom right) cells. **G,***SLFN11* WT, *SLFN11* KO, and *SLFN11* KO+SLFN11 U87 (left) and LN229 (right) cells were plated into round bottom 96-well plates under CSC culture conditions to form 3D neurospheres. After 7 days, spheres were imaged using a Cytation 3 Cell Imaging Multi-Mode Reader to determine cross-sectional area. Data are expressed as percentages of WT parental cells and represent means ± SEM of three independent experiments, each done in triplicate. Ordinary one-way ANOVA with Tukey multiple comparison test; *, *P* < 0.05; ***, *P <* 0.001.

To investigate the biological effects of *SLFN11* loss in GBM cells, CRISPR/Cas9-mediated gene KO was used to eliminate endogenous SLFN11 protein expression in U87 (Fig. 2A, left) and LN229 (Fig. 2A, right) GBM cell lines. The human *SLFN11* gene clusters together with *SLFN5*, *SLFN12*, *SLFN12L*, *SLFN13,* and *SLFN14* on chromosome 17 (11). Thus, modulating expression of one SLFN family member may alter expression of other SLFN family members in certain cellular contexts (17). While deletion of *SLFN11* resulted in some alterations in expression of *SLFN13* and *SLFN14*, these alterations were not consistent throughout cell lines, indicating the effects seen after *SLFN11* KO are specific (Supplementary Fig. S1). Loss of *SLFN11* significantly inhibited proliferation of KO cells *in vitro* (Fig. 2B) and reduced neurosphere formation as well as invasiveness in 3D cultures (Fig. 2C; Supplementary Fig. S2). We expanded our analysis to PDX cells which are known for improved preservation of patient tumor characteristics, relative to that of highly passaged GBM cell lines (34). CRISPR/Cas9-mediated KO of *SLFN11* in GBM6 PDX cells (Fig. 2D, left), significantly inhibited the ability of these cells to form neurospheres (Fig. 2D, right). To validate whether these growth inhibitory effects are indeed due to loss of *SLFN11*, we reexpressed *SLFN11* in U87 and LN229 *SLFN11* KO cells (Fig. 2E) and found that this reverted the antiproliferative effects to a level mirroring WT cells (Fig. 2F). Similar results were obtained when SLFN11 expression was rescued under 3D spheroid conditions (Fig. 2G). These results support the notion that elevated *SLFN11* expression promotes GBM cell proliferation and invasion and confirm these effects are specifically mediated by SLFN11.

### Loss of *SLFN11* Reduces Expression of Stem/Progenitor Markers

**FIGURE 3:**
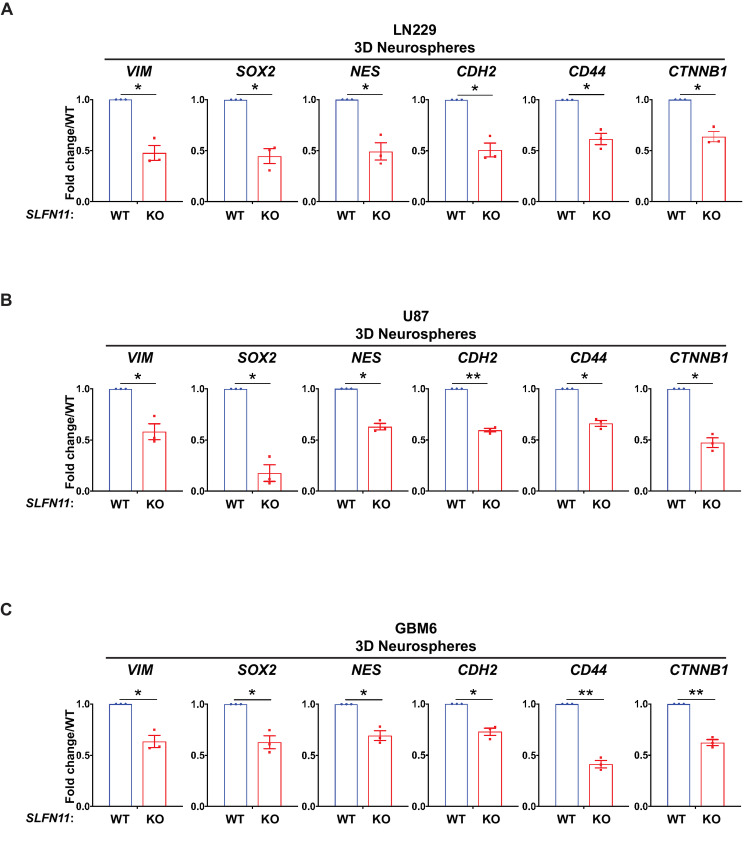
Loss of *SLFN11* downregulates expression of neural stem/progenitor marker genes. qRT-PCR analyses of relative mRNA expression of the indicated genes in *SLFN11* WT and *SLFN11* KO LN229 (**A**), U87 (**B**), and GBM6 (**C**) spheres are shown. The expression levels of the indicated genes were determined using *GAPDH* for normalization and as an internal control. The data are expressed as fold change over the corresponding WT spheres, and the graphs represent means ± SEM of three independent experiments. Two-tailed ratio paired *t* test; *, *P* < 0.05; **, *P* < 0.01.

Glioma stem cells (GSC) reside at the apex of GBM cellular hierarchy and contribute to long-term GBM progression, malignancy, and therapy resistance (35, 36). We investigated whether SLFN11 may modulate expression of genes associated with stem/progenitor markers. Expression data from cells grown in 3D as neurospheres under stem cell–permissive conditions revealed that KO of *SLFN11* in LN229 and U87 significantly reduced neural stem/progenitor cell marker expression including *VIM* (vimentin), *SOX2*, *NES* (nestin), *CDH2* (N-cadherin), *CD44*, and *CTNNB1* (β-catenin; Fig. 3A and B). In addition, we employed the PDX line GBM6 that has been shown to reflect GBM cellular heterogeneity including GSCs (8). Similar results were obtained for GBM6 PDX cells (Fig. 3C). Next, we employed our cell lines reexpressing SLFN11 (*SLFN11* KO+SLFN11) and observed increased expression of stem/progenitor markers (Supplementary Fig. S3), suggesting a partial rescue, and indicating the effects seen after *SLFN11* KO are specific.

### SLFN11 Associates with NFκB2/p100 and Stimulates Expression of NFκB Target Genes in GBM Cells

**FIGURE 4:**
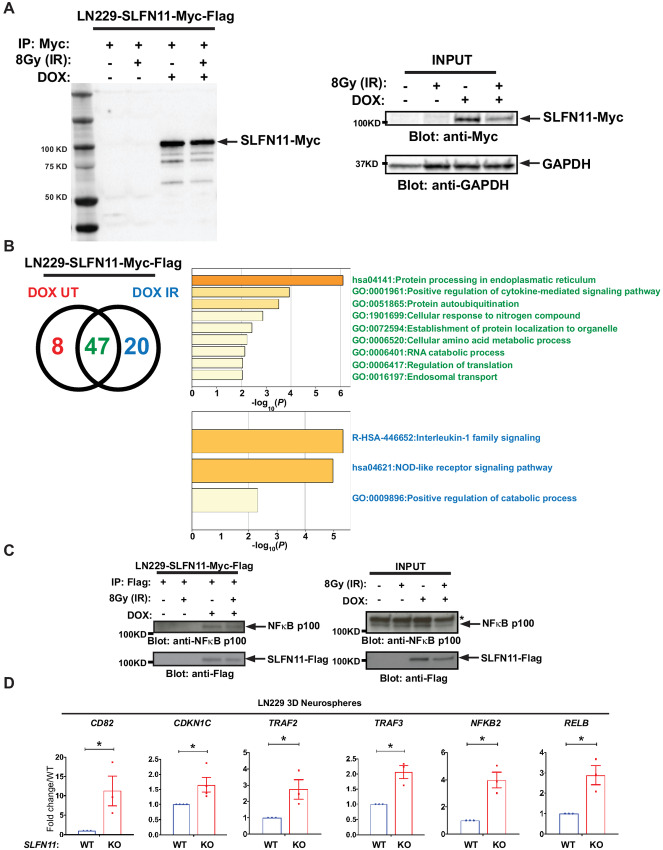
Identification of NFκB2 as a novel interactor of SLFN11. **A,** Stable LN229 DOX-inducible Myc-FLAG–tagged SLFN11-overexpressing cells (LN229-SLFN11-Myc-FLAG) were either left untreated (negative control) or were treated with DOX for 48 hours. Cells were then either left untreated or were irradiated (IR) with 8 Gy for 30 minutes, as indicated. Cell lysates were immunoprecipitated (IP) with Myc-conjugated sepharose beads. A total of 10% of the IP material was resolved by SDS-PAGE and immunoblotted with anti-Myc-HRP specific antibody (left). Equal amounts of cell lysates were resolved by SDS-PAGE and immunoblotted with the indicated antibodies (right). **B,** The remaining 90% of the IP material was submitted for LC/MS-MS analysis. Venn diagram shows the number of proteins identified as putative interactors of SLFN11 with and without IR (left). Ontology analysis of the 47 putative SLFN11 interactors identified independent from IR (top right). Ontology analysis of the 20 putative SLFN11 interactors identified under IR condition (bottom right). **C,** LN229-SLFN11-Myc-FLAG cells were either left untreated or treated with DOX for 48 hours and then either left untreated or were irradiated (IR) with 8 Gy for 30 minutes, as indicated. After cell lysis, protein-SLFN11-FLAG complexes were co-immunoprecipitated using FLAG antibody–conjugated sepharose beads followed by immunoblotting analysis using the indicated antibodies (left). Equal amounts of cell lysates from the co-IP experiment shown (INPUT) were resolved by SDS-PAGE and immunoblotted with the indicated antibodies (right). Asterisk indicates unspecific band. **D,** qRT-PCR analyses of relative mRNA expression of the indicated genes in *SLFN11* WT and *SLFN11* KO LN229 spheres are shown. The expression levels of the indicated genes were determined using *GAPDH* for normalization and as an internal control. The data are expressed as fold change over the corresponding WT spheres, and the graphs represent means ± SEM of four (*CDKN1C*) or three (*CD82, TRAF2, TRAF3, NFKB2, RELB*) independent experiments. Two-tailed ratio paired *t* test; *, *P* < 0.05.

To gain mechanistic insights into the pathways regulated by SLFN11 in GBM, lysates from LN229 cells expressing DOX-inducible Myc-tagged SLFN11 were incubated with anti-Myc antibody, and immunoprecipitates were analyzed using nano-LC/MS-MS. As SLFN11 is known to mediate responses to DNA damage (37), we also analyzed immunoprecipitates from cells treated with radiation. Myc-tagged SLFN11 was efficiently immunoprecipitated in lysates from DOX-induced LN229 cells treated with and without irradiation (Fig. 4A). Proteomic analyses identified 75 putative SLFN11 interacting proteins, 20 of which were discovered exclusively in irradiated cells and 8 exclusively in untreated cells (Fig. 4B, left; Supplementary Table S2). Ontology analysis of the 47 candidates found associated with SLFN11 regardless of treatment revealed that 15 of these genes are involved in the positive regulation of cytokine-mediated signaling pathways (Fig. 4B, top right panel in green; Supplementary Table S3). Of the 20 candidates associated with SLFN11 after cell irradiation, eight are known to be involved in IL1 family signaling (Fig. 4B, bottom panel in blue; Supplementary Table S4). Among these was the transcriptional regulator NFκB2 (Supplementary Table S4, highlighted in yellow). Changes in SLFN11 expression result in alterations of gene transcription (38). As NFκB2 represents a key transcription factor involved in various cellular responses, we sought to investigate the biological effects of this potential association in more detail. To corroborate this interaction, we used FLAG IP and found that under these conditions, SLFN11 associated with NFκB2 (NFκB2/p100) from LN229 cell lysates regardless of irradiation treatment (Fig. 4C). To investigate whether SLFN11 regulates NFκB transcriptional activity, we monitored mRNA levels of established NFκB target genes, such as *CD82*, *CDKN1C*, *TRAF2*, and *TRAF3* (39). As NFκB is known to be part of a positive regulatory feedback loop, we also investigated expression of *NFKB2* and *RELB* (39). We found that the transcript levels of all these NFκB target genes were significantly elevated in LN229 spheroids lacking SLFN11 (Fig. 4D). Our results suggest that SLFN11 physically associates with NFκB2/p100 in LN229 cells. Furthermore, loss of *SLFN11* triggers expression of numerous NFκB target genes in untreated cells, consistent with stimulation of NFκB transcriptional activity, independently of irradiation.

### Loss of *SLFN11* Promotes Transcriptional Activation of *CDKN1A* (p21) Through NFκB Noncanonical Signaling

**FIGURE 5:**
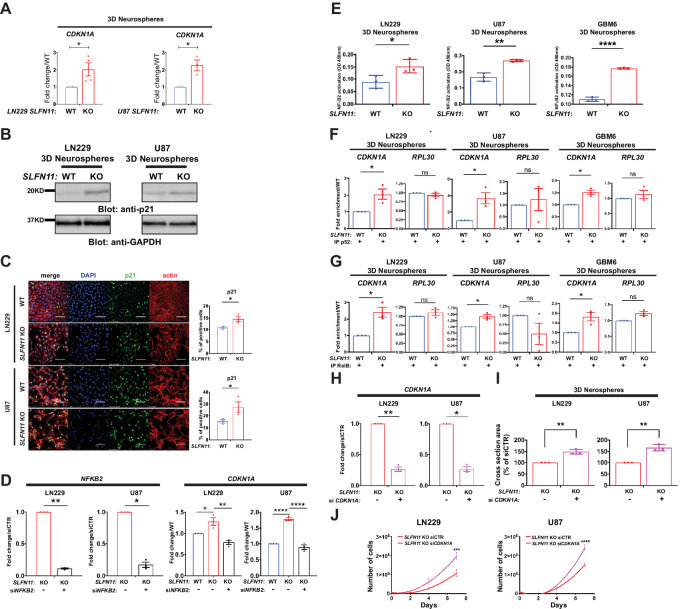
Loss of *SLFN11* stimulates NFκB2-dependent *CDKN1A* (p21) expression in GBM cells and enriched occupancy of p52 and RelB on the *CDKN1A* promoter. **A,** qRT-PCR analyses of the relative mRNA expression of *CDKN1A* in *SLFN11* WT and KO LN229 (left) and *SLFN11* WT and KO U87 (right) spheres are shown. The expression levels of *CDKN1A* were determined using *GAPDH* for normalization and as an internal control. The data are expressed as fold change over WT, and the graphs represent means ± SEM of three (U87) and four (LN229) independent experiments. Two-tailed ratio paired *t* test; *, *P* < 0.05. **B,***SLFN11* WT and KO LN229 (left) and U87 (right) cells were plated under CSC culture conditions to form 3D neurospheres. Equal amounts of total cell lysates from the indicated cells were resolved by SDS-PAGE and immunoblotted with the indicated antibodies. **C,***SLFN11* WT and KO LN229 (top left) and U87 (bottom left) cells were seeded onto coverslips. After 5 days, cells were fixed with 4% PFA and stained for DNA (blue), p21 (green), or actin (red). Representative images are shown (left). Corresponding WT and KO confocal microscopy images were acquired using identical settings. Scale bar, 100 μm. For quantification of p21-positive LN229 (top right) and U87 (bottom right) cells, pictures of four fields per sample were counted manually using ImageJ software. The data are expressed as percentage of p21-positive cells, and the graphs represent means ± SEM of three independent experiments. Two-tailed unpaired *t* test; *, *P* < 0.05. **D,***SLFN11* WT and KO LN229 and U87 cells were transfected with control siRNA and siRNA targeting *NFKB2* as indicated*.* Forty-eight hours after transfection, cells were collected for RNA isolation. qRT-PCR analysis of the relative mRNA expression of the indicated genes are shown. The expression levels of the indicated genes were determined using *GAPDH* for normalization and as an internal control. The data are expressed as fold change over WT samples, and the graphs represent means ± SEM of three independent experiments. qRT-PCR analysis for *NFKB2* is depicted in left panels. Two-tailed ratio paired *t* test; *, *P* < 0.05; **, *P* < 0.01. qRT-PCR analysis for *CDKN1A* is depicted in right panels. Ordinary one-way ANOVA with Tukey multiple comparison test; *, *P* < 0.05; **, *P* < 0.01; ***, *P <* 0.001; ****, *P* < 0.0001. **E,** NFκB2 ELISA activation assay. *SLFN11* WT and KO LN229, U87 and GBM6 cells were plated under CSC culture conditions to form 3D neurospheres. Cell lysates (30 μg) were assayed in a 96-well plate containing immobilized NFκB consensus site oligonucleotides. Subsequently, primary anti-p52 antibody was added, followed by detection with HRP secondary antibody at OD_450_ in a Cytation 3 Cell Imaging Multi-Mode Reader. The data are expressed as OD_450_, and the graphs represent means ± SEM of three independent experiments, each done in duplicate. Two-tailed unpaired *t* test; *, *P* < 0.05; **, *P* < 0.01; ****, *P* < 0.0001. ChIP for p52 (**F**) and RelB (**G**) in LN229 (left), U87 (middle), and GBM6 (right) spheres. *SLFN11* WT and KO LN229, U87 and GBM6 cells were plated under CSC culture conditions to form 3D neurospheres. After 7 days, cells were cross-linked with 1% formaldehyde. Chromatin–protein complexes were immunoprecipitated with anti-NFκB2 antibody (**F**) or anti-RelB antibody (**G**). Rabbit IgG antibody was used as a negative control. qPCR was performed on immunoprecipitated DNA with primers for the κB binding site in the *CDKN1A* promoter. Primers for the *RPL30* promoter were used as control. Data were normalized to their own IgG control and are expressed as fold enrichment over WT cells. Shown are means ± SEM of three independent experiments. Two-tailed ratio paired *t* test; *, *P* < 0.05. **H–J**, *SLFN11* KO LN229 and U87 cells were transfected with control siRNA and siRNA targeting *CDKN1A* as indicated*.* Twenty-four hours after transfection, cells were either collected for transcriptional analysis (**H**) or plated for neurosphere assays (**I**) and for proliferation assays (**J**). **H,** qRT-PCR analysis of the relative mRNA expression for *CDKN1A* is shown. The expression levels of the indicated genes were determined using *GAPDH* for normalization and as an internal control. The data are expressed as fold change over control siRNA samples, and the graphs represent means ± SEM of three independent experiments. Two-tailed ratio paired *t* test; *, *P* < 0.05; **, *P* < 0.01. **I,***SLFN11* KO siCTR and *SLFN11* KO si*CDKN1A* LN229 (left) and U87 (right) cells were plated into round bottom 96-well plates under CSC culture conditions to form 3D neurospheres. After 7 days, spheres were imaged using a Cytation 3 Cell Imaging Multi-Mode Reader to determine cross-sectional area. Data are expressed as percentages of siCTR cells and represent means ± SEM of three independent experiments, each done in triplicate. Two-tailed unpaired *t* test; **, *P* < 0.01. **J,***SLFN11* KO siCTR and *SLFN11* KO si*CDKN1A* LN229 (left) and U87 (right) cells were plated in 6-well plates and counted at days 2, 4, and 7 after seeding. Data are means of numbers of cells ± SEM of three independent experiments, each done in duplicate. Two-way ANOVA with Sidak multiple comparison test; ***, *P <* 0.001; ****, *P <* 0.0001.

Evidence indicates that NFκB can inhibit cell proliferation through induction of the cell-cycle inhibitor p21^CIP1^ (herein p21, encoded by *CDKN1A*) in certain cell types (40). qPCR and immunoblot results show that *CDKN1A* transcript and encoded protein are significantly elevated in LN229 and U87 *SLFN11* KO spheres (Fig. 5A and B). In addition, immunofluorescence analysis of cells lacking *SLFN11* revealed a significantly higher proportion of p21-positive cells (Fig. 5C). This indicates that KO of *SLFN11* stimulates induction of p21 protein expression in GBM cells. Next, we sought to determine whether the induction of p21 expression after loss of *SLFN11* is specifically dependent on NFκB2. Efficient knockdown of *NFKB2* by siRNA in *SLFN11*-deficient cells (Fig. 5D, left) blocked the increase in *CDKN1A* expression seen after loss of *SLFN11* (Fig. 5D, right). In addition, NFκB2 activity was significantly increased in *SLFN11* KO LN229, U87, and GBM6 neurospheres as indicated by ELISA assay results (Fig. 5E). To investigate the mechanism responsible for p21 induction by NFκB2 in *SLFN11*-deficient cells, we next performed ChIP experiments. We designed primers able to hybridize in the *CDKN1A* promoter region that contains the NFκB consensus DNA-binding sequence. In *SLFN11* KO neurospheres, we found a significant enrichment of p52 (the mature, activated NFκB2 form) occupancy on the *CDKN1A* promoter in LN229, U87, and GBM6 3D neurospheres (Fig. 5F). As activation of the noncanonical NFκB pathway triggers p52:RelB dimerization, we also assessed RelB in ChIP. Similar to p52, we detected significantly higher RelB occupancy at the *CDKN1A* promoter (Fig. 5G). We used the promoter for *RPL30* (encoding 60S ribosomal protein L30) as a control and found no significant enrichment of p52 or RelB occupancy on the *RPL30* promoter (Fig. 5F and G), indicating the enrichment on the *CDKN1A* promoter is specific. Together, these results indicate that *CDKN1A* is under the control of SLFN11-NFκB2 signaling in GBM and raise the possibility that p21 mediates, at least in part, the antineoplastic effects observed after *SLFN11* loss (see Fig. 2). In line with this, we found that efficient *CDKN1A* knockdown (Fig. 5H) restored neurosphere growth (Fig. 5I) and proliferation (Fig. 5J) in *SLFN11* KO LN229 and U87 cells.

### Loss of *SLFN11* Blocks Tumor Growth and Prolongs Survival in Mice Bearing GBM6 PDX

On the basis of the potent biological effects of *SLFN11* loss observed *in vitro*, we proceeded to examine KO effects on tumor cell growth *in vivo*.

**FIGURE 6:**
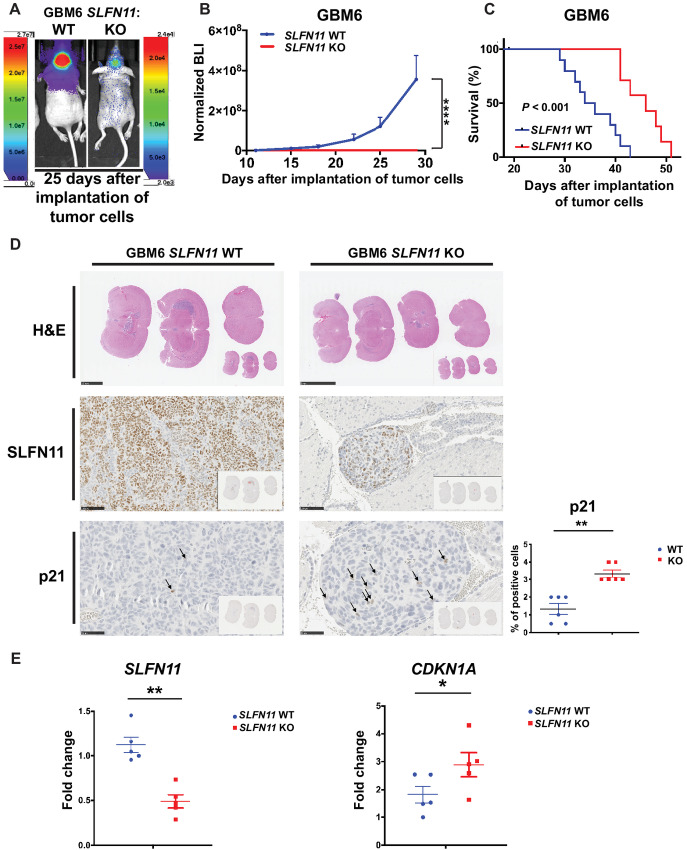
Loss of *SLFN11* inhibits tumor growth and prolongs survival in an intracranial GBM mouse model. **A–C,***SLFN11* WT (*n* = 10) and KO (*n* = 7) GBM6 luciferase-expressing cells (3 × 10^5^ cells/mouse) were injected into the brain of athymic NUDE mice and tumor growth was monitored weekly by bioluminescence imaging (BLI). **A,** Representative BLI of WT and *SLFN11* KO GBM6 tumors 25 days after implantation of tumor cells. **B,** Measurement of tumor volumes by BLI over time is shown. Data are means ± SEM of normalized BLI values for each genotypic group. Two-way ANOVA with Sidak multiple comparison test; ****, *P <* 0.001 for day 29. **C,** Kaplan–Meier survival curves of mice from experiment in **A** and **B**. Statistical analysis was performed using Kaplan–Meier with a Mantel–Cox (log-rank) test (*P <* 0.001). **D–F,***SLFN11* WT (*n* = 11) and KO (*n* = 11) GBM6 luciferase-expressing cells were injected into the brain of athymic NUDE mice and tumors were collected between days 16 and 20 after implantation of tumor cells. **D,** Representative images of H&E-stained mouse brains bearing WT (*n* = 6) or *SLFN11* KO (*n* = 6) GBM6 tumors (top). Scale bar, 2.5 mm. Representative SLNF11 immunostaining images of mouse brains bearing WT or *SLFN11* KO GBM6 tumors (second panels). Scale bar, 100 μm. Representative p21 immunostaining images of mouse brains bearing WT or *SLFN11* KO GBM6 tumors (bottom left). Scale bar, 50 μm*.* Percentage of p21-positive cells was quantified by a board-certified pathologist (bottom right). Data are expressed as percentage of positive cells and the graphs represent means ± SEM for each genotypic group. Mann–Whitney *U* test; **, *P* < 0.01. **E,** Expression of *SLFN11* and *CDKN1A* in orthotopic GBM6 tumors. Tumors were isolated from the brains of mice bearing WT (*n* = 5) or *SLFN11* KO (*n* = 5) GBM6 tumors. RNA was isolated and qRT-PCR was performed using primers for *SLFN11* (left) or *CDKN1A* (right). Expression level of the indicated genes was determined using *GAPDH* for normalization and as an internal control. Data are expressed as fold change over a randomly selected WT sample and the graphs represent means ± SEM for each genotypic group. Mann–Whitney *U* test; *, *P <* 0.05; **, *P* < 0.01.

In athymic mice intracranially implanted with GBM6-*SLFN11* KO cells, tumor growth was greatly inhibited as compared with mice that had received GBM6 WT cells (Fig. 6A and B). As a result, the lack of *SLFN11* prolonged survival (Fig. 6C). IHC analysis revealed that GBM6 WT tumors strongly expressed SLFN11, whereas GBM6-*SLFN11* KO tumors depicted greatly reduced SLFN11 staining (Fig. 6D and E, left). In agreement with our *in vitro* results (see Fig. 5), GBM6-*SLFN11* KO tumors exhibited significantly increased proportions of p21-positive cells (Fig. 6D, bottom) as well as increased expression of *CDKN1A* (Fig. 6E, right). In summary, loss of SLFN11 increases p21 expression, blocks tumor growth and prolongs survival in an intracranial PDX model.

## Discussion

The results of this study show that expression of *SLFN11* contributes to GBM growth and malignancy. Our investigation began with an analysis of TCGA data, which revealed that SLFN11 expression is inversely correlated with malignant glioma patient survival. This observation prompted our detailed examination of the molecular, cellular, and tumor biologic effects of SLFN11 in GBM. Genetic disruption of *SLFN11* in three distinct GBM cell sources inhibited cell proliferation and neurosphere growth, and reduced the expression of genes associated with progenitor/stem cell characteristics in neurosphere models, suggesting a GSC-supportive function of SLFN11. The potential role of SLFN11 in the regulation of stem cell properties is of particular interest given the importance of GSCs in contributing to GBM heterogeneity, response to treatment, and evolution (i.e., transcriptional subtype transitions; refs. 41–45).

Our results show that disruption of *SLFN11* expression greatly impairs tumor growth and significantly improved survival in an orthotopic PDX model. This finding is of utmost importance because SLFN11 mediates cell death in response to DNA-damaging agents (DDA) such as topoisomerase inhibitors and alkylating agents like cisplatin and TMZ (37, 46, 47). Hence, stimulation of *SLFN11* expression via promoter demethylation by histone deacetylase inhibitors has been suggested as a strategy to sensitize cancer cells to DDA (48). However, our findings necessitate careful assessment of such strategies in GBM because stimulation of *SLFN11* expression might trigger some undesired glioma-promoting effects. Supporting this notion are data from pediatric sarcomas including Ewing sarcoma, where SLFN11 is highly expressed. In these sarcomas, elevated SLFN11 protein expression was associated with worse outcome in terms of recurrence-free survival, and recurrent and resistant sarcomas still exhibited high *SLFN11* expression (24). Hence, in some cancers SLFN11 may execute additional roles, besides its DDA sensitizing ability, that may contribute to tumor progression.

SLFN11 is a well-established predictor of response to a variety of DDAs and PARP inhibitors (49). Still, whether SLFN11 expression may serve as a treatment biomarker in GBM remains to be elucidated. Our findings are consistent with SLFN11 as a potential prognostic biomarker for GBM. SLFN11 might also represent a potential target for therapeutic anti-GBM strategies with the caveat that SLFN11-depleted cells may exhibit reduced response to DNA damage induced by chemoradiation. Importantly, inhibition of ataxia telangiectasia and rad3-related (ATR) kinase was shown to reverse resistance in SLFN11-deficient cancer cells (21). Furthermore, a recent genome-wide RNAi chemosensitization screen identified several components of the ATR/CHK1 signaling pathway as potent hits in *SLFN11* KO cells and clinical inhibitors of these targets reversed the resistance to a broad range of DDAs seen in SLFN11-deficient cells (50). Thus, inhibitors of ATR pathway components might represent promising combinatorial candidates in SLFN11-depleted cells. Further studies are required to determine whether combined targeting of SLFN11 and components of the ATR/CHK1 pathway might enhance antitumor effects in patients with GBM treated with chemoradiation, the current standard of care.

Our data provide, for the first time, definitive evidence that SLFN11 associates with NFκB2 in GBM cells. The association of SLFN11 with NFκB2, appears to repress NFκB transcriptional activity because loss of SLFN11 stimulated expression of NFκB target genes. Mechanistically, loss of SLFN11 triggered enrichment of both p52 and RelB on the *CDKN1A* promoter and induced expression of p21 in an NFκB2-dependent way. Thus, based on these findings, it appears that SLFN11 blocks the p52:RelB heterodimer from occupying target gene promoters. On the basis of these findings, we propose a model in which SLFN11 associates with and inhibits NFκB2 to repress p21 in GBM. Consistent with this interpretation, p21 protein was enriched in orthotopic PDX tumors established from *SLFN11* KO cells. Importantly, knockdown of *CDKN1A* restored cell proliferation and neurosphere growth in *SLFN11* KO cells *in vitro* indicating the antineoplastic effects after *SLFN11* loss can be rescued by concomitant suppression of p21 expression. Together, these results provide compelling evidence for a SLFN11-NFκB2-p21 axis, in which SLFN11 suppresses NFκB2–mediated p21 expression, and by extension promotes GBM progression.

Irradiation and TMZ are essential components of the current treatment regimen for GBM, and both are potent DNA damage inducers. Besides p53, NFκB signaling is a major element for transcriptional reprogramming in response to DNA damage (51). DNA damage results in nuclear RelB enrichment and processing of p100 into p52, indicating a role also for the noncanonical NFκB pathway (51, 52). While SLFN11 is an established marker for sensitivity to DDA-mediated cancer cell killing, its role as a repressor of NFκB2 mediated transcription may complicate targeted approaches aiming to activate SLFN11 in GBM. Further studies are required to carefully dissect the effects of irradiation and TMZ on the transcriptional activity of SLFN11/NFκB2 associated signaling.

## Supplementary Material

Supplementary Figures S1-S3, Table S1 Fig. S1: Expression of SLFN family members after SLFN11 knockout. Fig. S2: Reduced 3-D invasion after SLFN11 knockout. Fig. S3: Expression of stem/progenitor markers after SLFN11 add-back. Table S1: Key resources table.

Supplementary Table S2 Supplementary Table S2 lists putative SLFN11 binding partners detected after untreated and/or irradiated cells.

Supplementary Table S3 Supplementary Table S3 lists proteins that were found to bind SLFN11 before or after irradiation.

Supplementary Table S4 Supplementary Table S4 lists proteins that were found to bind SLFN11 after irradiation.
