# Supplementary material for: SLFN11 Negatively Regulates Noncanonical NFκB Signaling to Promote Glioblastoma Progression
Source: Cancer Res Commun. 2022 Sep 13;2(9):966–78. doi: 10.1158/2767-9764.CRC-22-0192 (PMC9648417; doi:10.1158/2767-9764.CRC-22-0192)
Supplement: Supplementary Figures S1-S3, Table S1 — Fig. S1: Expression of SLFN family members after SLFN11 knockout. Fig. S2: Reduced 3-D invasion after SLFN11 knockout. Fig. S3: Expression of stem/progenitor markers after SLFN11 add-back. Table S1: Key resources table. [file crc-22-0192-s01.docx]

**Supplementary Information for**

**Schlafen 11 (SLFN11) negatively regulates non-canonical nuclear factor kappa B (NF-κB) signaling to promote glioblastoma progression.**

Mariafausta Fischietti^1,2,7^, Frank Eckerdt^1,2,3,7^, Ricardo E Perez^1,2^, Jamie N Guillen Magaña^1^, Candice Mazewski^1,2^, Sang Ho^1^, Christopher Gonzalez^1^, Lukas D Streich^4^, Elspeth M Beauchamp^1,2,5^, Amy Heimberger^3^, Aneta H Baran^1,2,5^, Feng Yue^1,6^, C. David James^1,3^, and Leonidas C. Platanias^1,2,5,^*^.^

Leonidas C. Platanias

Email: [l-platanias@northwestern.edu](mailto:l-platanias@northwestern.edu)

**This PDF file includes:**

Figures S1 to S3

Tables S1 to S4


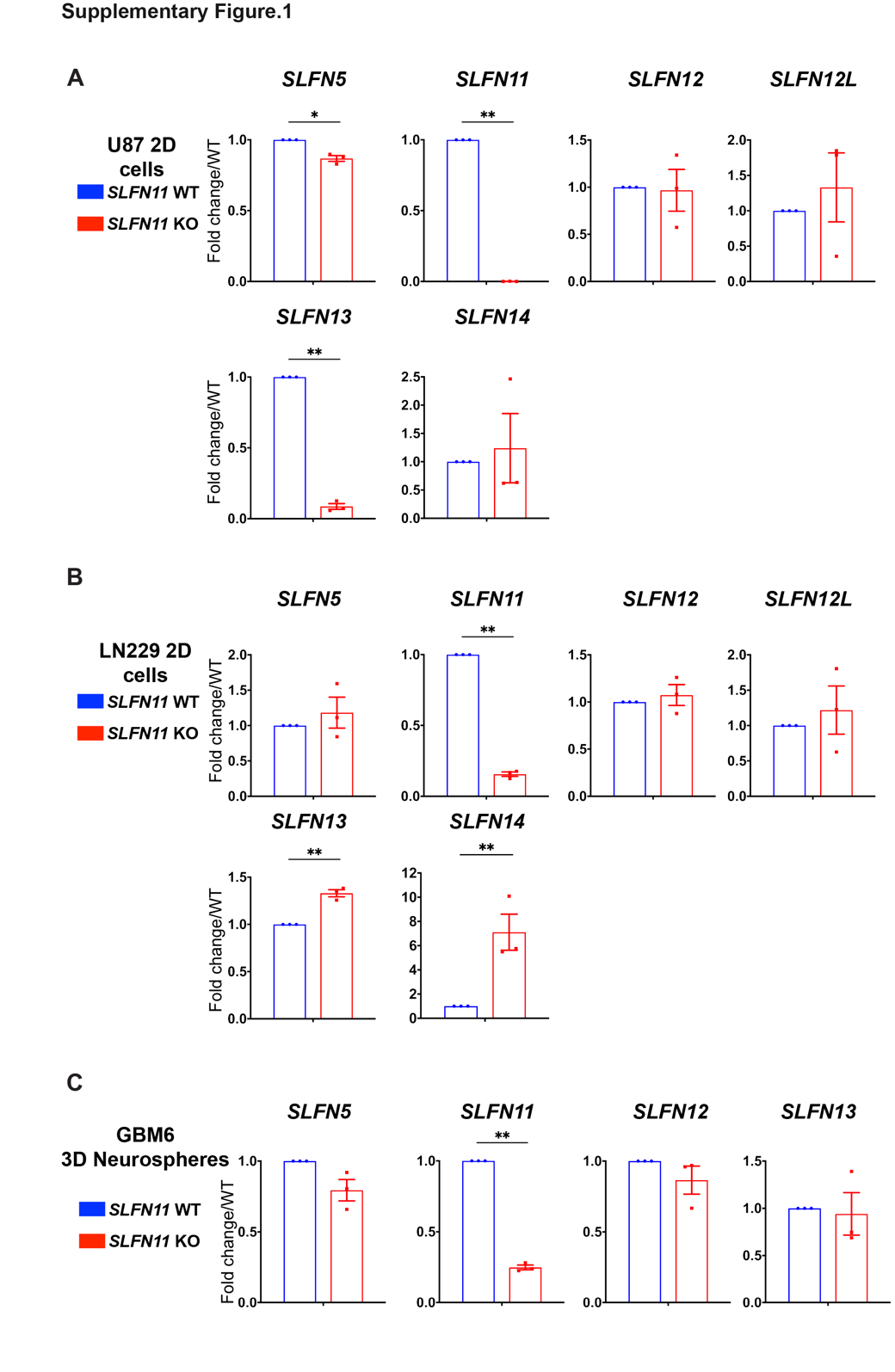


Fig. S1. Expression of SLFN family members in *SLFN11* WT and KO GBM cells and PDX neurospheres. (A-C) qRT-PCR analysis of the relative mRNA expression of the indicated genes for *SLFN11* WT and KO (A) U87 and (B) LN229 cells, and (C) GBM6 neurospheres is shown. For GBM6, CT values for SLFN12L and SLFN14 were below detection level. The expression level of the indicated genes was determined using *GAPDH* for normalization and as an internal control. Data are expressed as fold change over WT samples, and the graphs represent means ± SEM of three independent experiments. Two-tailed ratio paired *t* test; *, *p* < 0.05; **, *p* < 0.01.


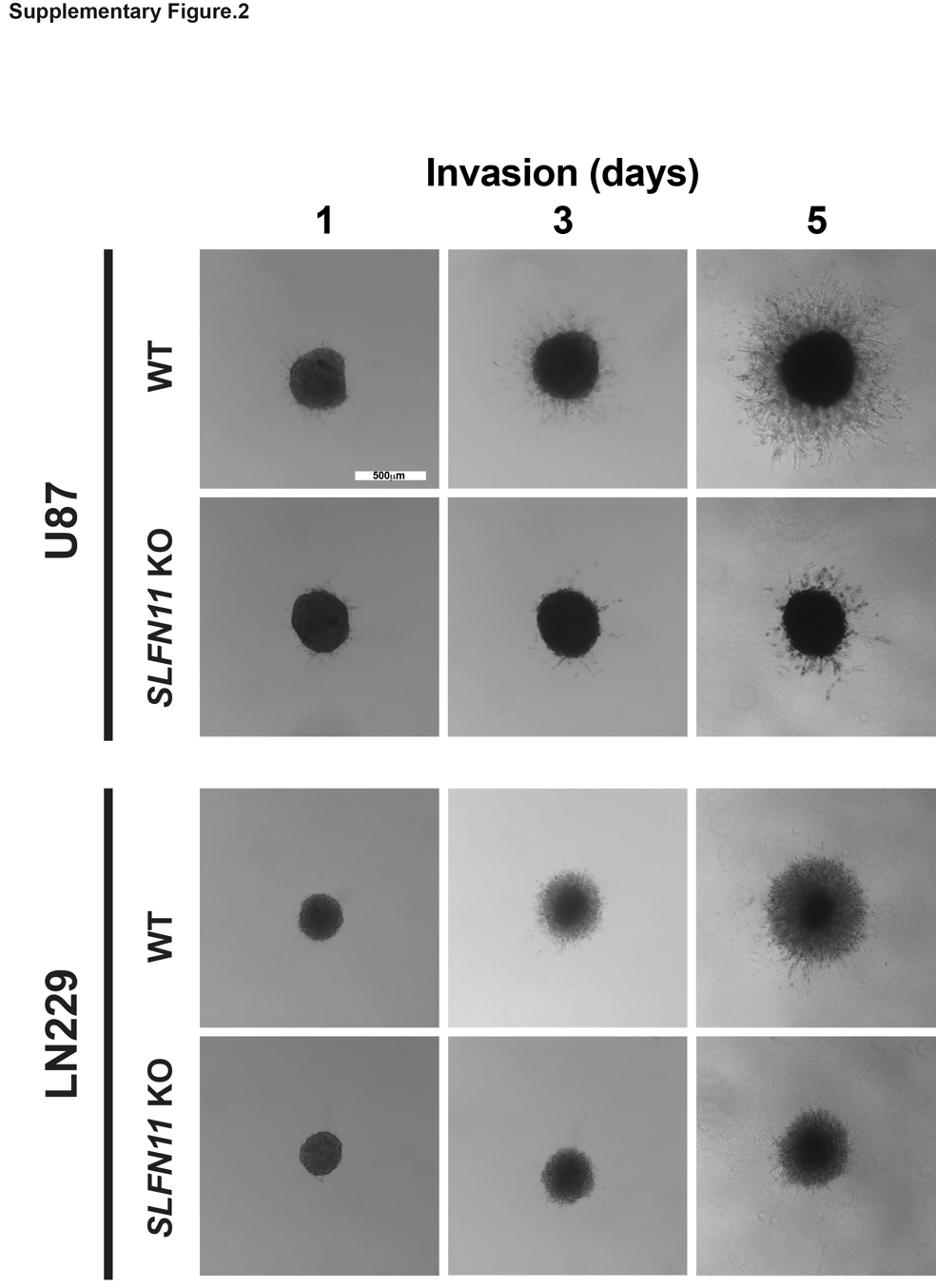


Fig. S2. **Loss of *SLFN11* reduces invasive properties of GBM cells in 3-D.** *SLFN11* WT and *SLFN11* KO U87 (upper panels) and LN229 (lower panels) cells were subjected to 3-D spheroid Matrigel invasion assays. Representative images (4 independent experiments, each done in triplicate) obtained 1, 3, and 5 days after seeding are shown.

**
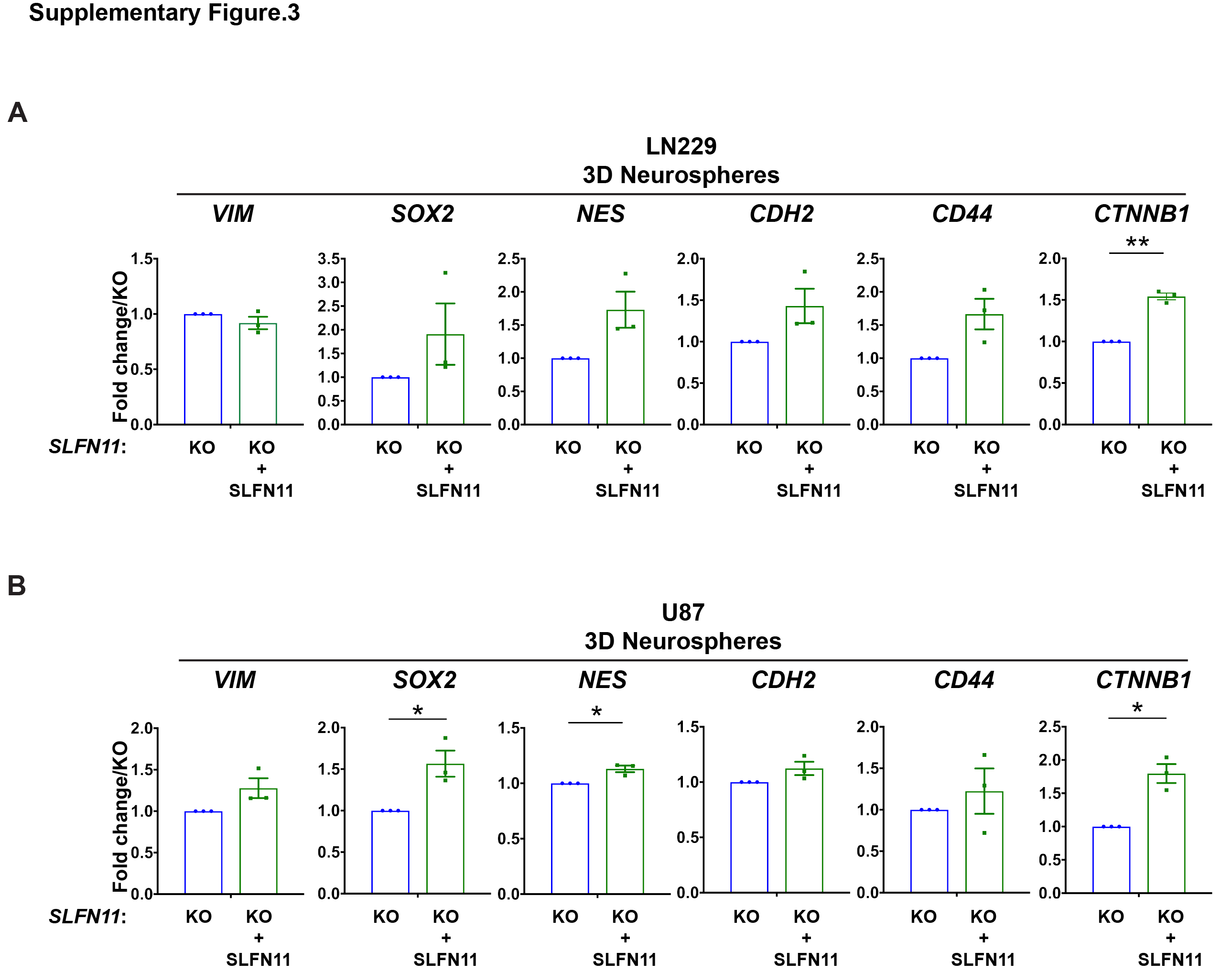
**

Fig. S3. **SLFN11 added back stimulates the expression of neural stem/progenitor marker genes.** (A-B) qRT-PCR analyses of relative mRNA expression of the indicated genes in *SLFN11* KO and *SLFN11* KO+SLFN11 LN229 (A) and U87 (B) spheres are shown. The expression levels of the indicated genes were determined using *GAPDH* for normalization and as an internal control. The data are expressed as fold change over the corresponding KO spheres, and the graphs represent means ± SEM of three independent experiments. Two-tailed ratio paired *t* test; *, *p* < 0.05; **, *p* < 0.01.

Table S1. Key resources table.

| REAGENT or RESOURCE | SOURCE | IDENTIFIER |
| --- | --- | --- |
| Antibodies | | |
| SLFN11 | Santa Cruz Biotechnology | Cat#: sc-515071  RRID:N/A |
| SLFN11 (D8W1B) | Cell Signaling | Cat#: 34858S  RRID:AB_2799063 |
| GAPDH | Millipore | Cat#: MAB374  RRID:AB_2107445 |
| FLAG-M2- HRP | Sigma-Aldrich | Cat#: A8592-0.2mg  RRID:AB_439702 |
| Myc- HRP | Cell Signaling Technology | Cat#: 2040S  RRID: AB_2148465 |
| NF-κB2 | Proteintech | Cat#: 10409-2-AP  RRID: AB_2151261 |
| NF-κB2 p100/p52 (D7A9K) | Cell Signaling Technology | Cat#: 37359S  RRID: AB_2799114 |
| RelB (D7D7W) | Cell Signaling Technology | Cat#: 10544S  RRID: AB_2797727 |
| p21 | Santa Cruz Biotechnology | Cat#: sc-397  RRID: AB_632126 |
| p21 | Cell Signaling Technology | Cat#: 2947S  RRID: AB_823586 |
| Mouse Anti-rabbit IgG (Conformation Specific) (L27A9) mAb (HRP Conjugate) | Cell Signaling Technology | Cat#: 5127  RRID:AB_10892860 |
| Rabbit IgG HRP Linked Whole Ab | GE Healthcare | NA934-1ML  RRID:AB_772206 |
| Goat Anti-Mouse IgG (H + L)-HRP Conjugate | Biorad | Cat#:1706516  RRID:AB_11125547 |
| Chemicals, Peptides, and Recombinant Proteins | | |
| Doxycycline hyclate | Sigma Aldrich | Cat#: D9891-5G |
| Acridine orange solution | Sigma Aldrich | Cat#: A9231 |
| Goat anti-Rabbit IgG (H+L) Highly Cross-Adsorbed Secondary Antibody, Alexa Fluor 488 | Thermo Fisher Scientific | Cat# A11034  RRID:AB_2576217 |
| Alex Fluor 546 Phalloidin | Thermo Fisher Scientific | Cat# A22283 |
| ProLong™ Gold Antifade Mountant | Thermo Fisher Scientific | Cat# P36934 |
| Myc-Tag (9B11) Mouse mAb (Sepharose^®^ Bead Conjugate) | Cell Signaling | Cat# 3400  RRID:AB_10692357 |
| Anti-FLAG M2 affinity gel | Sigma Aldrich | Cat# A2220-5ML  RRID:AB_10063035 |
| D-Luciferin 1 g/ Potassium Salt | GoldBio | Cat#: LUCK-1G |
| DAPI | Roche | Cat#: 10236276001 |
| GE Healthcare Amersham™ ECL™ | GE Healthcare | Cat#: 45-000-875 |
| Lenti-X GoStick | Clontech | Cat#: 631280 |
| Lenti-X Concentrator | Clontech | Cat#: 631231 |
| TransDux MAX Lentivirus Transduction enhancer | System Biosciences | Cat#: LV860A-1 |
| Lipofectamine™ RNAiMAX Transfection Reagent | Thermo Fisher | Cat#: 133778-150 |
| Turbofect | Thermo Fisher | Cat#: R0531 |
| Critical Commercial Assays | | |
| RNeasy Plus Mini Kit | Qiagen | Cat#: 74136 |
| SimpleChIP^®^ Enzymatic Chromatin IP Kit (Magnetic Beads) | Cell Signaling Technology | Cat#: 9003 |
| NF-κB Family Transcriprion Factor Assay Kit | Active Motif | Cat#: 43296 |
| SsoAdvanced™ Universal Probes Supermix, 1,000 x 20 µl rxns, 10 ml (10 x 1 ml) | Biorad | Cat#: 172-25282 |
| SsoAdvanced™ Universal SYBR® Green Supermix | Biorad | Cat#: 172-5271 |
| Experimental Models: Cell Lines and reagents | | |
| LN229 | Dr. Shi-Yuan Cheng | N/A |
| U87 | Dr. Alexander Stegh | N/A |
| GBM6 | Dr. Charles D. James | N/A |
| Hygromycin B (50mg/ml) | Thermo Fisher | Cat#: 10687010 |
| Opti-MEM | Thermo Fisher | Cat#: 31985-070 |
| 293T | Clontech | Cat#: 632180  RRID:CVCL_0063 |
| TrypLE Express | Thermo Fisher | Cat#: 12604-013 |
| DMEM/F12 | Thermo Fisher | Cat#: 10565-042 |
| Recombinant Human FGF-basic (154 a.a.) | Peprotech | Cat#: 100-18B |
| Epidermal Growth Factor, Urogastrone, URG | Peprotech | Cat#: AF-100-15 |
| Heparin sodium salt | Sigma-Aldrich | Cat#: H3149-10KU |
| B27® Serum-Free Supplement | Thermo Fisher | Cat#: 17504-044 |
| Gentamycin | Sigma-Aldrich | Cat#: G1397-10ml |
| BD Matrigel, Growth factor–reduced (GFR), Phenol Red and LDEV-Free | Thermo Fisher | Cat#: 356231 |
| Puromycin | Thermo Fisher | Cat#: A11138-03 |
| 96-well round bottom ultra-low attachment plate | Fisher-Corning | Cat#: 07-20-680 |
| 10 µL Microliter Syringe Model 701 N, Cemented Needle, 26s gauge, 2 in, point style 2 | Hamilton | Cat#: 80300 |
| Experimental Models: Organisms/Strains | | |
| NCRNU-F sp/sp (NCr*-Foxn1^nu^)* nude mouse | Taconic | Cat#: TAC:ncrnu  RRID:IMSR_TAC:ncrnu |
| Oligonucleotides/Taqman Probes | | |
| VIMENTIN Taqman Primer | Thermo Fisher | Hs00185584_m1 |
| SOX2 Taqman Primer | Thermo Fisher | Hs01053049_s1 |
| CDH2 Taqman Primer | Thermo Fisher | Hs00983056_m1 |
| CD44 Taqman Primer | Thermo Fisher | Hs01075861_m1 |
| CTNNB1 Taqman Primer | Thermo Fisher | Hs0035049_m1 |
| NES Taqman Primer | Thermo Fisher | Hs04187831_g1 |
| CD82 Taqman Primer | Thermo Fisher | Hs01017982_m1 |
| CDKN1C Taqman Primer | Thermo Fisher | Hs00175938_m1 |
| TRAF2 Taqman Primer | Thermo Fisher | Hs00184192_m1 |
| TRAF3 Taqman Primer | Thermo Fisher | Hs00936781_m1 |
| NFκB2 Taqman Primer | Thermo Fisher | Hs01028901_g1 |
| RelB Taqman Primer | Thermo Fisher | Hs00232399_m1 |
| IL6Taqman Primer | Thermo Fisher | Hs00174131_m1 |
| IL1β Taqman Primer | Thermo Fisher | Hs01555410_m1 |
| CXCL8 Taqman Primer | Thermo Fisher | Hs00174103_m1 |
| CDKN1A Taqman Primer | Thermo Fisher | Hs00355782_m1 |
| SLFNL1 Taqman Primer | Thermo Fisher | Hs00379087_m1 |
| SLFN5 Taqman Primer | Thermo Fisher | Hs01072905_g1 |
| SLFN11 Taqman Primer | Thermo Fisher | Hs00536981_m1 |
| SLFN12 Taqman Primer | Thermo Fisher | Hs01049939_m1 |
| SLFN12L Taqman Primer | Thermo Fisher | Hs04334089_m1 |
| SLFN13 Taqman Primer | Thermo Fisher | Hs00431187_m1 |
| SLFN14 Taqman Primer | Thermo Fisher | Hs00976826_m1 |
| GAPDH Taqman Primer | Thermo Fisher | Hs03929097_g1 |
| ChIP CDKN1A FW  5’-CTATCTTTGTTCCGCCTCTTC-3’ | IDT | N/A |
| ChIP CDKN1A REV  5’-TGGGAACAATGTCACCATTT-3’ | IDT | N/A |
| Recombinant DNA | | |
| SLFN11 HDR Plasmid (h2) | Santa Cruz Biotechnology | Cat#: sc-401137-HDR-2 |
| SLFN11 CRISPR/Cas9 KO Plasmid (h2) | Santa Cruz Biotechnology | Cat#: sc-401137-KO-2 |
| pLVX-hEF1α-Cas9-Blast | Horizon | Cat#: CAS10138 |
| pLVX-CMV-SLFN11 sgRNAs-PURO | Horizon | Cat#: GSGH11838 |
| pLVX-tetON-SLFN11-MYC-FLAG | BioInnovatise, Inc | N/A |
| ORF expression clone for SLFN11(NM_001104589.1) (Purified plasmid) | GeneCopoeia | Cat#: EX-H3535-M12 |
| pLenti-CMV Hygro Dest (W117-1) | Addgene | Cat#: 17423  RRID:Addgene_17423 |
| pENTR4-FLAG (w210-2) | Addgene | Cat#: 17423  RRID:Addgene_17423 |
| ON-TARGETplus Human NFKB2 (4791) siRNA - SMARTpool, 5 nmol | Dharmacon | Cat#: L-003918-00-0005 |
| ON-TARGETplus Non-targeting Pool, 20 nmol | Dharmacon | Cat#: D-001810-10-20 |
| p21 siRNA (h) | Santa Cruz Biotechnology | Cat#:SC-29427 |
| [Control siRNA-](https://www.scbt.com/p/control-sirna-h)H | Santa Cruz Biotechnology | Cat#:SC-44236 |
| Lenti-X™ Packaging Single Shots (VSV-G) | Takara-Clontech | Cat#: 631282 |
| Software and Algorithms | | |
| Prism | Graphpad | N/A  RRID:SCR_002798 |
| Excel | Microsoft | N/A |
| Scaffold 4 | Proteome Software | N/A  RRID:SCR_014345 |
| Metascape | Metascape.org | http://metascape.org |
| Jaspar | Castro-Mondragon JA et al., 2022 | N/A |
| Fiji-ImageJ | Schindelin et al., 2012 | N/A |
| Adobe Illustrator | Adobe | N/A |
| NDP.view2 | Hamamatsu | U12388-01 |

Table S2 (separate file). Putative SLFN11 binding partners detected after untreated and/or irradiated cells.

Table S3 (separate file). Gene ontology of the proteins that bind SLFN11 before or after irradiation.

Table S4 (separate file). Gene ontology of the proteins that bind SLFN11 after irradiation.
